# Association among Polymorphisms in EGFR Gene Exons, Lifestyle and Risk of Gastric Cancer with Gender Differences in Chinese Han Subjects

**DOI:** 10.1371/journal.pone.0059254

**Published:** 2013-03-29

**Authors:** Junfeng Zhang, Zhen Zhan, Juan Wu, Chunbing Zhang, Yaping Yang, Shujuan Tong, Zheng Sun, Lei Qin, Xuewen Yang, Wei Dong

**Affiliations:** 1 Discipline of Chinese and Western Intergrative Medicine, College of Basic Medicine, Nanjing University of Chinese Medicine, Nanjing, China; 2 Department of Clinical Laboratory, First Affiliated Hospital of Nanjing University of Chinese Medicine, Nanjing, China; 3 Gastrointestinal Tumor Surgery, First Affiliated Hospital of Nanjing University of Chinese Medicine, Nanjing, China; The Liverpool Cancer Research UK Centre, United Kingdom

## Abstract

**Background:**

The epidermal growth factor receptor (EGFR) gene plays a key role in tumor survival, invasion, angiogenesis, and metastatic spread. Recent studies showed that gastric cancer (GC) was associated with polymorphisms of the EGFR gene and environmental influences, such as lifestyle factors. In this study, seven known SNPs in EGFR exons were investigated in a high-risk Chinese population in Jiangsu province to test whether genetic variants of EGFR exons and lifestyle are associated with an increased risk of GC.

**Methodology/Principal Findings:**

A hospital-based case-control study was performed in Jiangsu province. The results showed that smoking, drinking and preference for salty food were significantly associated with the risk of GC. The differences of lifestyle between males and females might be as the reason of higher incidence rates in males than those in females. Seven exon SNPs were genotyped rs2227983,rs2072454,rs17337023,rs1050171,rs1140475, rs2293347, and rs28384375. It was noted that the variant rs2072454 T allele and TT genotype were significantly associated with an increased risk of GC. Interestingly, our result suggested the ACAGCA haplotype might be associated with decreased risk of GC. However, no significant association was examined between the other six SNPs and the risk of GC both in the total population and the age-matching population even with gender differences.

**Conclusions:**

Smoking, drinking and preference for salty food were significantly associated with the risk of GC in Jiangsu province with gender differences. Although only one SNP (rs2072454) was significantly associated with an increased risk of GC, combined the six EGFR exon SNPs together may be useful for predicting the risk of GC.

## Introduction

Although both the incidence and mortality of gastric cancer (GC) have declined in recent years, GC was the fourth most common malignancy in the world in 2008, with approximately 989,600 new cases. Men generally develop GC twice as frequently as women and about 72% of new cases occur in developing countries. In general, the highest incidence rates are in Asia, particularly in East Asian countries such as Korea, Japan, and China [1].

Indeed, almost 40% of all GC cases occur in China, and there is a remarkable geographical variation in GC rates throughout China [2]. More than two-thirds of the patients diagnosed with GC in China have unresectable disease and a median survival of six to nine months. Moreover, in patients with resectable tumors, the local and distant recurrence rates are high, and the 5-year survival rate is less than 30% [3]. Sun *et al.* [4] assessed the impact of GC on the Chinese population by epidemiological analysis of its mortality distribution from 1990 to 1992, the results showed that GC was the leading cause of cancer-related mortality in China.

It is now generally accepted that the pathogenesis of GC involves a multi-factorial interaction between environmental triggers and genetic susceptibility. Epidemiological studies have identified age, gender differences and a number of environmental factors that may contribute to the development of GC, including a salty diet, tobacco smoking, alcohol consumption and *Helicobacter pylori* infection [5]–[8]. Other studies have identified host factors and genetic alterations that also play an important role in the development and progression of GC through gene-environment interactions [9], [10].

The epidermal growth factor receptor (EGFR) gene is located in the short arm of human chromosome 7 and produces a glycoprotein with a molecular weight of 170 kDa that has a high affinity for its ligands, including epidermal growth factor (EGF) and transforming growth factor alpha (TGF-α). EGFR participates in several essential tumorigenic mechanisms, such as tumor survival, invasion, angiogenesis, and metastatic spread. EGFR expression has been observed in numerous human tumors, and several studies demonstrated that overexpression of EGFR correlates with a poor outcome [11]. In GC, EGFR overexpression correlates with advanced tumor stage and a poor clinical outcome [12]. However, the roles that EGFR overexpression and genetic alterations play in gastric carcinogenesis remain unclear. Moreover, only a few single nucleotide polymorphisms (SNPs) have been found to associate with GC development and outcome [11], [13].

In this study, we hypothesized that the environmental exposures and gender differences act as effect modifiers on a background of genetic variation in EGFR exons which may affect EGFR function, thereby shaping GC susceptibility. To test this hypothesis, a hospital-based study was performed in which 387 GC cases and 392 cancer-free controls in a high-risk Chinese population were genotyped for seven known SNPs in EGFR exons, namely, rs2227983 A>G, rs2072454 C>T, rs17337023 A>T, rs1050171 G>A, rs1140475 C>T,rs2293347 G>A, and rs28384375 T>C.

## Materials and Methods

### Recruitment of Cases and Control Participants

Initially, 401GC cases and 420 controls were identified; 3 cases of cancer lack of questionnaires as well as 11 tumors other than adenocarcinoma were excluded; 18 controls were excluded by immoderate serum cancer-related biomarkers and 10 controls were excluded by geographical deviation. Overall, a total population with 387 cases and 392 controls were available for the current study on the basis of prospective power analyses, it is a pity that controls were about 10 years younger than cases, thus, an age-matching population with 294 cases and 294 controls was extracted from the total population for the collation of age-matching. All subjects were genetically unrelated ethnic Han Chinese. The patients with primary GC were recruited from the Department of Surgical Gastroenterology in the Jiangsu Provence Hospital of Traditional Chinese Medicine (TCM) between January, 2008 and July, 2010 in Nanjing city, Jiangsu province. The cancer-free healthy controls were consecutively recruited from Jiangsu Hospital of TCM, and they were hospital visitors for an annual check-up during the same period. To be included in the study, the patients (a) males or females over 20 years old but under 80 years old, (b) had to be of Han Chinese ethnicity (self-reported), (c) came from three regions of Jiangsu province and had to be a local resident for at least 5 years, (d) had to have newly histopathologically diagnosed primary GC, (e) had to lack previous malignant tumors in other organs, and (f) had not had antitumor therapy before recruitment, including chemotherapy and radiotherapy. Trained interviewers used a pre-tested questionnaire to collect epidemiological data from the participants, namely, demographic factors such as age and gender, and known risk factors for GC (such as tobacco smoking, alcohol consumption, and a family history of digestive tract cancer). Individuals who smoked one or more times a day for over a year were defined as smokers, and those who consumed three or more alcoholic drinks a week for over 6 months were considered to be chronic drinkers [14], [15]. After signing informed consent forms, each subject donated 3–5 ml of peripheral blood to be used for genomic DNA extraction. The research protocol was approved by the institutional review board of Jiangsu Provence Hospital of TCM.

### Genomic DNA Isolation from Peripheral Blood Cells

A commercial blood DNA extraction kit (AxyPrep-96 kit, Axygen, CA, USA) was used to extract genomic DNA from the blood samples. The purified DNAs were stored at −20°C until they were used for genotype testing. The quality of DNA was assessed by agarose gel electrophoresis.

### Genotyping

Polymerase chain reaction-ligation detection reaction (PCR-LDR) methods were used for genotyping [16]. Primers were synthesized by Shanghai Sangon Biological Engineering Technology and Services (No.698, Xiangmin Rd, Songjiang District, Shanghai, China). Each set of ligase detection reaction probes comprised one common probe and two discriminating probes for the two types (Table S1).

The target DNA sequences were amplified using a multiplex PCR method. PCRs for each subject were carried out in a final volume of 20 µl containing 1 × PCR buffer, 3.0 mmol/l MgCl_2_, 2.0 mmol/l deoxynucleotide triphosphates, 1 µl primers, 0.2 µl QIAGEN HotStarTaq Polymerase (QIAGEN, China), 4 µl of 1 × Q-solution, and 10–20 ng genomic DNA. Thermal cycling was performed for five SNPs (rs2227983, rs17337023, rs1140475, rs2293347 and rs2072454) in the Gene Amp PCR system 9600 (PerkinElmer) with an initial denaturation for 15 min at 95°C, followed by 35 cycles of denaturation at 94°C for 30 s, annealing at 59°C for 1 min, and extension at 72°C for 1 min, followed by a final extension at 72°C for 7 min. The protocol for rs28384375 amplification consisted of an initial denaturation for 15 min at 95°C, followed by 35 cycles of denaturation at 94°C for 30 s, annealing at 56°C for 1 min, and extension at 72°C for 1 min, followed by a final extension at 72°C for 7 min. The protocol for rs1050171 consisted of an initial denaturation for 15 min at 95°C, followed by 35 cycles of denaturation at 94°C for 30 s, annealing at 53°C for 1 min, and extension at 72°C for 1 min, followed by a final extension at 72°C for 7 min.

The ligation reaction for each subject was carried out in a final volume of 10 µl containing 1 × NEB Taq DNA ligase buffer, 12.5 pmol of each probe mix, 0.05 µl Taq DNA ligase [NEB Biotechnology (Beijing)], and 1 µl of multi-PCR product. The probe sequences are shown in Table S2. In total, 35 cycles consisting of 95°C for 2 min, 94°C for 30 s, and 60°C for 2 min were performed. The fluorescent products of the ligase detection reactions were differentiated by an ABI sequencer 377 (Figure S1). To confirm the accuracy of the PCR-LDR genotyping method, direct DNA sequencing of randomly selected PCR products was performed. The proportion of the sequencing samples was about 5%. The PCR-LDR genotyping results showed complete agreement with the direct DNA sequencing results.

### Statistical Analyses

All statistical analyses were conducted by using SPSS software version 16.0 (SPSS Inc., Chicago, IL, USA). To compare the observed genotype frequencies in the control group with the expected frequencies, the Hardy-Weinberg equilibrium (HWE) was tested by a goodness-of-fit χ^2^-test. Cases and controls were compared in terms of demographic characteristics, lifestyle factors, and the allele frequencies of each SNP using the *χ^2^*-test. For each polymorphism, odds ratios (OR) and 95% confidence intervals (CI) were calculated from conditional logistic regression models to estimate the main effect of each polymorphism with GC while adjusting for continuous age, gender and lifestyle factors. Logistic regression analyses using the major allele as a reference were employed to estimate adjusted ORs, 95% CIs, and *P* values. However, we found that the gender and age variable did not conform to the assumption of proportionality. Thus, gender stratification and age-matching were used to measure ORs and 95% CIs. For lifestyle variables that showed significant relations with GC in analyses controlled for matching factors only, we further assessed their independence in analyses adjusted for additional potential confounders (i.e., smoking, drinking, salty food, eating time and eating breakfast) in the age-matching population. And PHASE software package version 2.0 was used to infer haplotype frequencies based on the observed EGFR genotypes. All *P* values were two-sided and a *P* value <0.05 was considered statistically significant.

## Results

### Characteristics of the Study Subjects

The characteristics of these study subjects were consistent with previously described [17], [18]. In this paper, the total population with 387 cases and 392 controls and an age-matching population with 294 cases and 294 controls were included in the current analyses. Gender, age, and geographic region distribution of study subjects are shown in Table 1.

**Table 1:** Demographic characteristics of subjects.

	Total population		Age-matching population	
	GC cases	Controls	GC cases	Controls
**Number**	387	392	294	294
**Age**	59.4±13.2	50.6±11.7	55.30±9.62	54.70±11.01
*P*	0.001^a^		0.067^a^	
**Gender**				
Male	264 (68.2)	237 (60.5)	188 (63.9)	161 (54.8)
Female	123 (31.8)	155 (39.5)	106 (36.1)	133 (45.2)
*P*	0.005^b^		0.023^b^	
**Geographic regions**				
Nanjing	342 (88.4)	347 (88.5)	262 (89.1)	267 (90.8)
Danyang	37 (9.6)	37 (9.4)	25 (8.5)	19 (6.5)
Yancheng	8 (2.0)	8 (2.0)	7 (2.4)	8 (2.7)

a Non-parametric test.

b Two-sided test.

To examine the differences in GC incidence between males and females, the distributions of select lifestyle variables were analyzed with gender differences. The results showed that six lifestyle factors (i.e., regularly taking meals, preference for salty food, eating time, smoking status, drinking status, and eating breakfast) were significantly correlated to the risk of GC, and their effects might be modified by gender and age. Four lifestyle factors, i.e., preference for salty food, eating time, smoking status and drinking status, were significantly different between males and females (*P*<0.05) (Table 2). It was noted that the percentages of the males carrying unhealthy dietary habits, such as preference for salty food, short eating time, smoking and drinking, were significantly higher than those of the females (*P*<0.05), which might be as the reason of higher incidence rates in males than those in females. In the total population, regularly taking meals and eating breakfast decreased the risk of female GC, while drinking could increase the risk of male GC (Table 3). In addition, both preference for salty food and smoking increased the risk of male and female GC (*P*<0.05). Similar results were also found after age-matching that regularly taking meals decreased the risk of GC and preference for salty food and smoking increased the risk of both male and female GC (*P*<0.05, Table 4). However, there were two exceptions, that was drinking increased the risk of male GC (*P*<0.05) but not female GC (*P*>0.05) perhaps due to the limited female population, while daily eating breakfast decreased the risk of female GC (*P*<0.05) but not male GC (*P*>0.05) (Table 4).

**Table 2:** Differences of select lifestyle variables between males and females.

Variable	Total population			Age-matching population		
	Male	Female	*P* *	Male	Female	*P* *
	n = 500	n = 279		n = 349	n = 239	
	N (%)	N (%)		N (%)	N (%)	
**Regularly taking meals**						
Often	413 (81.3)	227 (83.8)	0.150	284 (81.4)	199 (83.3)	0.085
Occasionally	54 (10.6)	32 (11.8)		35 (10.0)	30 (12.6)	
Seldom	41 (8.1)	12 (4.4)		30 (8.6)	10 (4.2)	
**Preference for salty food**						
Salty	216 (42.7)	88 (32.7)	0.022	153 (44.0)	78 (32.8)	0.023
Average	201 (39.7)	121 (45.0)		128 (36.8)	108 (45.4)	
Not well salted	89 (17.6)	60 (22.3)		67 (19.3)	52 (21.8)	
**Eating time**						
<10 min	301 (59.3)	99 (36.5)	<0.001	204 (58.5)	88 (36.8)	<0.001
10 ∼ 20 min	186 (36.6)	150 (55.4)		134 (38.4)	133 (55.6)	
>20 min	21 (4.1)	22 (8.1)		11 (3.2)	18 (7.5)	
**Smoking status**						
Never	205 (40.4)	264 (97.4)	<0.001	138 (39.5)	232 (97.1)	<0.001
Ever	303 (59.6)	7 (2.6)		211 (60.5)	7 (2.9)	
**Drinking status**						
Never	172 (33.9)	237 (87.5)	<0.001	118 (33.8)	209 (87.4)	<0.001
Seldom	160 (31.5)	29 (10.7)		98 (28.1)	26 (10.9)	
Often	82 (16.1)	3 (1.1)		58 (16.6)	2 (0.8)	
Daily	94 (18.5)	2 (0.7)		75 (21.5)	2 (0.8)	
**Eating breakfast**						
Never	9 (2.0)	3 (1.2)	0.812	6 (2.0)	2 (0.9)	0.721
Seldom	18 (3.9)	12 (4.9)		13 (4.3)	12 (5.6)	
Often	48 (10.5)	24 (9.8)		30 (9.8)	21 (9.7)	
Daily	381 (83.6)	207 (84.1)		256 (83.9)	181 (83.8)	

* Two-sided test.

**Table 3:** Distributions of select lifestyle variables in the total population with gender differences.

Variable	Total population			Males			Females		
	Cases	Controls	*P* *	Cases	Controls	*P* *	Cases	Controls	*P* *
	n = 387	n = 392		n = 264	n = 237		n = 123	n = 155	
	N (%)	N (%)		N (%)	N (%)		N (%)	N (%)	
**Regularly taking meals**									
Often	304 (78.6)	336 (85.7)	0.010	216 (79.7)	197 (83.1)	0.536	88 (75.9)	139 (89.7)	0.001
Occasionally	47 (12.1)	39 (9.9)		30 (11.1)	24 (10.1)		14.7 (17)	15 (9.7)	
Seldom	36 (9.3)	17 (4.3)		25 (9.2)	16 (6.8)		11 (9.5)	1 (0.6)	
**Preference for salty food**									
Salty	203 (52.5)	101 (26.0)	<0.001	144 (53.1)	72 (30.6)	<0.001	59 (50.9)	29 (19.0)	<0.001
Average	147 (38.0)	175 (45.1)		103 (38.0)	98 (41.7)		44 (37.9)	77 (50.3)	
Not well salted	37 (9.6)	112 (28.9)		24 (8.9)	65 (27.7)		13 (11.2)	47 (30.7)	
**Eating time**									
<10 min	212 (54.8)	188 (48.0)	0.136	164 (60.5)	137 (57.8)	0.823	48 (41.4)	51 (32.9)	0.263
10 ∼ 20 min	157 (40.6)	179 (45.7)		96 (35.4)	90 (38.0)		61 (52.6)	89 (57.4)	
>20 min	18 (4.7)	25 (6.4)		11 (4.1)	10 (4.2)		7 (6.0)	15 (9.7)	
**Smoking status**									
Never	204 (52.7)	265 (67.6)	<0.001	94 (34.7)	111 (46.8)	0.005	110 (94.8)	154 (99.4)	0.020
Ever	183 (47.3)	127 (32.4)		177 (65.3)	126 (53.2)		6 (5.2)	1 (0.6)	
**Drinking status**									
Never	185 (47.8)	224 (57.1)	0.001	89 (32.8)	83 (35.0)	0.007	96 (82.8)	141 (91.0)	0.239
Seldom	89 (23.0)	100 (25.5)		72 (26.6)	88 (37.1)		17 (14.7)	12 (7.7)	
Often	49 (12.7)	36 (9.2)		47 (17.3)	35 (14.8)		2 (1.7)	1 (0.6)	
Daily	64 (16.5)	32 (8.2)		63 (23.2)	31 (13.1)		1 (0.9)	1 (0.6)	
**Eating breakfast**									
Never	6 (1.9)	6 (1.5)	0.001	3 (1.4)	6 (2.6)	0.159	3 (3.2)	0 (0.0)	<0.001
Seldom	21 (6.7)	9 (2.3)		11 (5.0)	7 (3.0)		10 (10.8)	2 (1.3)	
Often	42 (13.4)	30 (7.7)		29 (13.1)	19 (8.1)		13 (14.0)	11 (7.2)	
Daily	245 (78.0)	343 (88.4)		178 (80.5)	203 (86.4)		67 (72.0)	140 (91.5)	

* Two-sided test.

**Table 4:** Distributions of select lifestyle variables in the age-matching population with gender differences.

Variable	Age-matching population			Males			Females		
	Cases	Controls	*P* *	Cases	Controls	*P* *	Cases	Controls	*P* *
	n = 294	n = 294		n = 188	n = 161		n = 106	n = 133	
	N (%)	N (%)		N (%)	N (%)		N (%)	N (%)	
**Regularly taking meals**									
Often	224 (76.2)	259 (88.1)	<0.001	144 (76.6)	140 (81.4)	0.044	80 (75.5)	119 (89.5)	0.003
Occasionally	40 (13.6)	25 (8.5)		23 (12.2)	12 (10.0)		17 (16.0)	13 (9.8)	
Seldom	30 (10.2)	10 (3.4)		21 (11.2)	9 (8.6)		9 (8.5)	1 (0.8)	
**Preference for salty food**									
Salty	161 (54.8)	70 (24.0)	<0.001	106 (56.4)	47 (29.4)	<0.001	55 (51.9)	23 (17.4)	<0.001
Average	104 (35.4)	132 (45.2)		64 (34.0)	64 (40.0)		40 (37.7)	68 (51.5)	
Not well salted	29 (9.9)	90 (30.8)		18 (9.6)	49 (30.6)		11 (10.4)	41 (31.1)	
**Eating time**									
<10 min	159 (54.1)	133 (45.2)	0.100	115 (61.2)	89 (55.3)	0.352	44 (41.5)	44 (33.1)	0.317
10 ∼ 20 min	122 (41.5)	145 (49.3)		66 (35.1)	68 (42.2)		56 (52.8)	77 (57.9)	
>20 min	13 (4.4)	16 (5.4)		7 (3.7)	4 (2.5)		6 (5.7)	12 (9.0)	
**Smoking status**									
Never	165 (56.1)	205 (69.7)	0.001	65 (34.6)	73 (45.3)	0.040	100 (94.3)	132 (99.2)	0.025
Ever	129 (43.9)	89 (30.3)		123 (65.4)	88 (54.7)		6 (5.7)	1 (0.8)	
**Drinking status**									
Never	147 (50.0)	180 (61.2)	0.006	60 (31.9)	58 (36.0)	0.041	87 (82.1)	122 (91.7)	0.148
Seldom	61 (20.7)	63 (21.4)		44 (23.4)	54 (33.5)		17 (16.0)	9 (6.8)	
Often	37 (12.6)	23 (7.8)		36 (19.1)	22 (13.7)		1 (0.9)	1 (0.8)	
Daily	49 (16.7)	28 (9.5)		48 (25.5)	27 (16.8)		1 (0.9)	1 (0.8)	
**Eating breakfast**									
Never	5 (2.2)	3 (1.0)	<0.001	3 (2.1)	3 (1.9)	0.051	2 (2.4)	0 (0.0)	0.001
Seldom	19 (8.2)	6 (2.1)		9 (6.2)	4 (2.5)		10 (11.8)	2 (1.5)	
Often	31 (13.4)	20 (6.9)		20 (13.7)	10 (6.3)		11 (12.9)	10 (7.6)	
Daily	176 (76.2)	261 (90.0)		114 (78.1)	142 (89.3)		62 (72.9)	119 (90.8)	

* Two-sided test.

### Genotyping Distribution and Risk of GC

In the present study, only the T allele of the missense locus rs28384375 was detected. Regarding the six remaining SNPs, the observed genotype frequencies in the controls were in HWE. Since no statistical significance was lost, only the adjusted statistical results were shown in the tables. In the total population, the association among the distribution of EGFR gene alleles, genotypes and the risk of GC was shown in Table 5. Only a slight difference was observed in terms of the allelic distribution of rs2072454 (*χ^2^* = 3.844, *P = *0.050), and logistic regression analyses revealed that the variant of this allele may be associated with GC risk (adjusted OR = 1.23, 95% CI = 1.00–1.50). However, no significant differences between cases and controls in terms of allele distribution were observed with gender differences. The rs2293347 polymorphism was associated with male GC cases because 73.0% of GC cases carried a G allele compared with 29.5% of health controls (adjusted OR = 6.45, 95% CI = 4.89–8.51, *P*<0.001), but no genotypes were significantly associated with the risk of GC. In the contrary female population the GA genotype could decrease the risk of female GC because of 34.5% of GC cases carrying a GA genotype compared with 38.9% of health controls, but no significant differences between the distribution of allele and genotype frequencies (*P*>0.05). In addition, no significant differences were examined at the other four SNPs (*P*>0.05), i.e. rs2227983, rs17337023, rs1050171 and rs1140475 between cases and controls even with gender differences.

**Table 5:** Association between EGFR exon polymorphisms and the risk of GC in the total population with gender differences.

SNPs	Total population			Males			Females		
	Cases	Controls	OR (95% CI)^b^	Cases	Controls	OR (95% CI)^c^	Cases	Controls	OR (95% CI)^c^
	n = 387	n = 392		n = 271	n = 237		n = 116	n = 155	
	N (%)	N (%)		N (%)	N (%)		N (%)	N (%)	
**rs2227983**	N = 371	N = 386	*P* a * = *0.481	N = 259	N = 234	*P* a * = *0.402	N = 112	N = 152	*P* a * = *0.932
AA	109 (29.4)	116 (30.1)	1.00	85 (32.8)	81 (33.7)	1.00	24 (21.4)	35 (23.0)	1.00
GA	187 (50.4)	205 (53.1)	0.89 (0.39–2.08)	116 (44.8)	112 (46.2)	3.47 (1.01–11.98)	71 (63.4)	93 (61.2)	0.48 (0.07–3.44)
GG	75 (20.2)	65 (16.8)	0.92 (0.50–1.70)	58 (22.4)	41 (20.1)	1.66 (0.73–3.80)	17 (15.2)	24 (15.8)	0.56 (0.11–2.84)
GA/GG	262 (70.6)	270 (69.9)	0.93 (0.51–1.71)	174 (67.2)	153 (65.4)	2.01 (0.92–4.42)	88 (78.6)	117 (77.0)	0.55 (0.11–2.69)
Allele			*P* a * = *0.428			*P* a * = *0.291			*P* a * = *0.911
A	405 (54.6)	437 (56.6)	1.00	286 (55.2)	274 (58.6)	1.00	119 (53.1)	163 (53.6)	1.00
G	337 (45.4)	335 (43.4)	1.09 (0.89–1.33)	232 (44.8)	194 (41.4)	0.87 (0.68–1.12)	105 (46.9)	141 (46.4)	0.98 (0.69–1.39)
**rs2072454**	N = 384	N = 390	*P* a * = *0.038	N = 268	N = 237	*P* a * = *0.050	N = 116	N = 153	*P* a * = *0.510
CC	130 (33.9)	144 (36.9)	1.00	97 (36.2)	91 (38.4)	1.00	33 (28.4)	53 (34.6)	1.00
TC	180 (46.9)	197 (50.5)	0.98 (0.61–1.57)	114 (42.5)	115 (48.5)	0.98 (0.44–2.17)	66 (56.9)	82 (53.6)	1.07 (0.34–3.36)
TT	74 (19.3)	49 (12.6)	1.55 (0.80–3.00)	57 (21.3)	31 (13.1)	5.12 (1.04–25.10)	17 (14.7)	18 (11.8)	3.69 (0.39–34.79)
TC/TT	254 (66.1)	246 (63.1)	1.02 (0.65–1.60)	171 (63.8)	146 (61.6)	1.32 (0.61–2.87)	83 (71.6)	100 (65.4)	0.78 (0.25–2.40)
Allele			*P* a * = *0.050			*P* a * = *0.093			*P* a * = *0.288
C	440 (57.3)	485 (62.2)	1.00	308 (57.5)	297 (62.7)	1.00	132 (56.9)	188 (61.4)	1.00
T	328 (42.7)	295 (37.8)	1.23 (1.00–1.50)	228 (42.5)	177 (37.3)	0.81 (0.63–1.04)	100 (43.1)	118 (38.6)	0.83 (0.59–1.17)
**rs17337023**	N = 382	N = 390	*P* a * = *0.290	N = 268	N = 237	*P* a * = *0.441	N = 114	N = 153	*P* a * = *0.816
AA	123 (32.2)	133 (34.1)	1.00	92 (34.3)	88 (37.1)	1.00	31 (27.2)	45 (29.4)	1.00
TA	171 (44.8)	185 (47.4)	1.02 (0.59–1.76)	109 (40.7)	101 (42.6)	1.25 (0.56–2.79)	62 (54.4)	84 (54.9)	0.58 (0.16–2.07)
TT	88 (23.0)	72 (18.5)	0.95 (0.44–2.06)	67 (25.0)	48(20.3)	5.47 (1.44–20.76)	21 (18.4)	24 (15.7)	1.46 (0.23–9.14)
TA/TT	259 (67.8)	257 (65.9)	1.08 (0.63–1.84)	176 (65.7)	149 (62.9)	1.86 (0.87–3.96)	83 (72.8)	108 (70.6)	0.72 (0.21–2.46)
Allele			*P* a = 0.200			*P* a = 0.227			*P* a = 0.569
A	417 (54.6)	451 (57.8)	1.00	293 (54.7)	277 (58.4)	1.00	124 (54.4)	174 (56.9)	1.00
T	347 (45.4)	329 (42.2)	1.14 (0.93–1.40)	243 (45.3)	197 (41.6)	0.86 (0.67–1.10)	104 (45.6)	132 (43.1)	0.91 (0.64–1.28)
**rs1050171**	N = 365	N = 379	*P* a * = *0.211	N = 254	N = 230	*P* a * = *0.641	N = 111	N = 149	*P* a * = *0.057
GG	269 (73.7)	257 (67.8)	1.00	185 (72.8)	163 (70.9)	1.00	84 (75.7)	94 (63.1)	1.00
GA	84 (23.0)	107 (28.2)	1.02 (0.42–2.50)	61 (24.0)	56 (24.3)	7.35 (0.91–59.51)	23 (20.7)	51 (34.2)	0.00 (0.00–)
AA	12 (3.3)	15 (4.0)	0.81 (0.54–1.21)	8 (3.1)	11 (4.8)	2.31 (0.40–13.26)	4 (3.6)	4 (2.7)	0.00 (0.00–)
GA/AA	96 (26.3)	122 (32.2)	0.80 (0.55–1.14)	246 (96.9)	219 (95.2)	2.73 (0.48–15.65)	27 (24.3)	55 (36.9)	4.84E8 (0.00–)
Allele			*P* a * = *0.088			*P* a * = *0.446			*P* a * = *0.082
G	622 (85.2)	621 (81.9)	1.00	431 (84.8)	382 (83.0)	1.00	191 (86.0)	239 (80.2)	1.00
A	108 (14.8)	137 (18.1)	0.79 (0.60–1.04)	77 (15.2)	78 (17.0)	0.88 (0.62–1.23)	31 (14.0)	59 (19.8)	1.52 (0.95–2.45)
**rs1140475**	N = 383	N = 389	*P* a * = *0.848	N = 267	N = 236	*P* a * = *0.676	N = 116	N = 153	*P* a * = *0.779
CC	351 (91.6)	355 (91.3)	1.00	246 (92.1)	215 (91.1)	1.00	105 (90.5)	140 (91.5)	1.00
TC	32 (8.4)	34 (8.7)	1.20 (0.64–2.24)	21 (7.9)	21 (8.9)	3.00 (0.37–24.17)	11 (9.5)	13 (8.5)	0.39 (0.09–1.77)
Allele			*P* a * = *0.852			*P* a * = *0.683			*P* a * = *0.784
C	734 (95.8)	744 (95.6)	1.00	513 (96.1)	451 (95.6)	1.00	221 (95.3)	293 (95.8)	1.00
T	32 (4.2)	34 (4.4)	0.95 (0.58–1.56)	21 (3.9)	21 (4.4)	1.14 (0.61–2.11)	11 (4.7)	13 (4.2)	0.89 (0.39–2.03)
**rs2293347**	N = 374	N = 381	*P* a * = *0.855	N = 261	N = 232	*P* a * = *0.310	N = 261	N = 232	*P* a * = *0.474
GG	203 (54.3)	204 (53.5)	1.00	140 (53.6)	27 (11.6)	1.00	63 (55.8)	82 (55.0)	1.00
GA	140 (37.4)	141 (37.0)	1.11 (0.49–2.51)	101 (38.7)	83 (35.8)	0.32 (0.10–1.02)	39 (34.5)	58 (38.9)	4.31E8 (0.00–)
AA	31 (8.3)	36 (9.4)	1.13 (0.72–1.76)	20 (7.7)	122 (52.6)	0.80 (0.35–1.86)	11 (9.7)	9 (6.0)	0.59 (0.17–2.00)
GA/AA	171 (45.7)	177 (46.5)	1.06 (0.71–1.57)	121 (46.4)	205 (88.4)	0.64 (0.30–1.39)	50 (44.2)	67 (45.0)	0.83 (0.25–2.75)
Allele			*P* a * = *0.680			*P* a <0.001			*P* a * = *0.701
G	546 (73.0)	549 (72.0)	1.00	381 (73.0)	137 (29.5)	1.00	165 (73.0)	222 (74.5)	1.00
A	202 (27.0)	213 (28.0)	0.95 (0.76–1.20)	141 (27.0)	327 (70.5)	6.45 (4.89–8.51)	61 (27.0)	76 (25.5)	0.93 (0.63–1.37)

a Two-sided test;

b ORs were adjusted for age, gender and lifestyle factors;

c ORs were adjusted for age and lifestyle factors.

In the age-matching population, the association among the distribution of EGFR gene alleles, haplotypes and the risk of GC was shown in Table 6. A significant difference was observed in terms of the allelic distribution (*χ^2^* = 4.795, *P = *0.029) and genotype distribution (*χ^2^* = 6.668, *P = *0.036) of rs2072454, other than the results in Table 5. In addition, logistic regression analyses revealed that the variant of rs2072454 T allele could increase the risk of GC (adjusted OR = 0.77, 95% CI = 0.61–0.97; *P*<0.05). According to the genotype distribution, a significant difference was also observed in the male population (*χ^2^* = 7.914, *P = *0.019) rather than female population (*χ^2^* = 0.452, *P = *0.798). However, there were no significant differences between cases and controls even with gender differences for the remaining SNPs, namely, rs2227983, rs17337023, rs1140475, rs1050171 and rs2293347 (*P*>0.05).

**Table 6:** Association between EGFR exon polymorphisms and the risk of GC in the age-matching population with gender differences.

SNPs	Age-matching population			Males			Females		
	Cases	Controls	OR (95% CI)^b^	Cases	Controls	OR (95% CI)^c^	Cases	Controls	OR (95% CI)^c^
	n = 294	n = 294		n = 188	n = 161				
	N (%)	N (%)		N (%)	N (%)		N (%)	N (%)	
**rs2227983**	N = 284	N = 288	*P* a * = *0.855	N = 182	N = 158	*P* a * = *0.613	N = 102	N = 130	*P* a * = *0.900
AA	72 (25.4)	77 (26.7)	1.00	50 (27.5)	50 (31.6)	1.00	22 (21.6)	27 (20.8)	1.00
GA	155 (54.6)	158 (54.9)	3.01 (0.92–9.82)	90 (49.5)	77 (48.7)	11.91 (1.45–98.07)	65 (63.7)	81 (62.3)	0.33 (0.05–2.35)
GG	57 (20.1)	53 (18.4)	1.38 (0.68–2.83)	42 (23.1)	31 (19.6)	1.98 (0.81–4.83)	15 (14.7)	22 (16.9)	0.45 (0.09–2.27)
GA/GG	212 (74.7)	211 (73.3)	1.60 (0.80–3.21)	132 (72.5)	108 (68.4)	2.69 (1.13–6.38)	80 (78.4)	103 (79.2)	0.43 (0.09–2.07)
Allele			*P* a * = *0.605			*P* a * = *0.320			*P* a * = *0.747
A	299 (52.6)	312 (54.2)	1.00	190 (52.2)	177 (56.0)	1.00	109 (53.4)	135 (51.9)	1.00
G	269 (47.4)	264 (45.8)	0.94 (0.75–1.19)	174 (47.8)	139 (44.0)	0.86 (0.63–1.16)	95 (46.6)	125 (48.1)	1.06(0.74–1.53)
**rs2072454**	N = 292	N = 292	*P* a * = *0.036	N = 186	N = 161	*P* a * = *0.019	N = 106	N = 131	*P* a * = *0.798
CC	86 (29.5)	100 (34.2)	1.00	57 (30.6)	59 (36.6)	1.00	29 (27.4)	41 (31.3)	1.00
TC	145 (49.7)	154 (52.7)	1.27 (0.65–2.50)	84 (45.2)	82 (50.9)	1.29 (0.54–3.06)	61 (57.5)	72 (55.0)	1.07 (0.35–3.33)
TT	61 (20.9)	38 (13.0)	13.48 (1.73–105.1)	45 (24.2)	20 (12.4)	5.41E8 (0.00–)	16 (15.1)	18 (13.7)	3.70 (0.40–34.60)
TC/TT	206 (70.6)	192 (65.7)	1.72 (0.89–3.34)	129 (69.5)	102 (63.3)	1.91 (0.82–4.45)	77 (72.6)	90 (68.7)	1.29 (0.43–3.92)
Allele			*P* a * = *0.029			*P* a * = *0.018			*P* a * = *0.562
C	317 (54.3)	354 (60.6)	1.00	198 (53.2)	200 (62.1)	1.00	119 (56.1)	154 (58.8)	1.00
T	267 (45.7)	230 (39.4)	0.77 (0.61–0.97)	174 (46.8)	122 (37.9)	0.69 (0.51–0.94)	93 (43.9)	108 (41.2)	0.90 (0.62–1.29)
**rs17337023**	N = 291	N = 292	*P* a * = *0.472	N = 186	N = 161	*P* a * = *0.530	N = 105	N = 131	*P* a * = *0.961
AA	86 (29.6)	92 (31.5)	1.00	59 (31.7)	57 (35.4)	1.00	27 (25.7)	35 (26.7)	1.00
TA	135 (46.4)	142 (48.6)	1.22 (0.62–2.41)	76 (40.9)	68 (42.2)	1.59 (0.66–3.81)	59 (56.2)	74 (56.5)	0.61 (0.17–2.15)
TT	70 (24.1)	58 (19.9)	5.19 (1.442–18.66)	51 (27.4)	36 (22.4)	14.03 (1.74–112.88)	19 (18.1)	22 (16.8)	1.21 (0.20–7.57)
TA/TT	205 (70.5)	200 (68.5)	1.65 (0.85–3.18)	127 (68.3)	104 (64.6)	2.49 (1.08–5.74)	78 (74.3)	96 (73.2)	0.70 (0.21–2.40)
Allele			*P* a * = *0.292			*P* a * = *0.249			*P* a * = *0.803
A	307 (52.7)	326 (55.8)	1.00	194 (52.2)	182 (56.5)	1.00	113 (53.8)	144 (55.0)	1.00
T	275 (47.3)	258 (44.3)	0.88 (0.70–1.11)	178 (47.8)	140 (43.5)	0.84 (0.62–1.13)	97 (46.19)	118 (45.0)	0.96 (0.66–1.37)
**rs1050171**	N = 278	N = 283	*P* a * = *0.205	N = 176	N = 156	*P* a * = *0.060	N = 102	N = 127	*P* a * = *0.126
GG	207 (74.5)	195 (68.9)	1.00	130 (73.9)	112 (71.8)	1.00	77 (75.5)	83 (65.4)	1.00
GA	65 (23.4)	76 (26.9)	5.11 (0.61–42.65)	44 (25.0)	35 (22.4)	6.71E10 (0.00−)	21 (20.6)	41 (32.3)	0.00 (0.00−)
AA	6 (2.2)	12 (4.2)	3.80 (0.51–28.40)	2 (1.1)	9 (5.8)	9.11E9 (0.00−)	4 (3.9)	3 (2.4)	0.00 (0.00−)
GA/AA	71 (25.5)	88 (31.1)	0.89 (0.41–1.94)	46 (26.1)	44 (28.2)	2.42 (0.67–8.72)	25 (24.51)	44 (34.6)	0.50 (0.16–1.54)
Allele			*P* a * = *0.079			*P* a * = *0.230			*P* a * = *0.220
G	479 (86.2)	466 (82.3)	1.00	304 (86.4)	259 (83.0)	1.00	175 (85.78)	207 (81.5)	1.00
A	77 (13.8)	100 (17.7)	1.34 (0.97–1.85)	48 (13.6)	53 (17.0)	1.30 (0.85–1.98)	29 (14.22)	47 (18.5)	1.37 (0.83–2.27)
**rs1140475**	N = 291	N = 291	*P* a * = *0.648	N = 185	N = 160	*P* a * = *0.427	N = 106	N = 131	*P* a * = *0.780
CC	269 (92.4)	266 (91.4)	1.00	173 (93.5)	146 (91.2)	1.00	96 (90.6)	120 (91.6)	1.00
TC	22 (7.6)	25 (8.6)	0.86 (0.27–2.76)	12 (6.5)	14 (8.8)	2.22 (0.26–18.74)	10 (9.4)	11 (8.4)	0.35 (0.08–1.65)
Allele			*P* a * = *0.655			*P* a * = *0.436			*P* a * = *0.785
C	560 (96.2)	557 (95.7)	1.00	358 (96.8)	306 (95.6)	1.00	202 (95.28)	251 (95.8)	1.00
T	22 (3.8)	25 (4.3)	1.14 (0.64–2.05)	12 (3.2)	14 (4.4)	1.37 (0.62–3.00)	10 (4.72)	11 (4.2)	0.89 (0.37–2.13)
**rs2293347**	N = 285	N = 286	*P* a * = *0.706	N = 181	N = 159	*P* a * = *0.126	N = 104	N = 127	*P* a * = *0.481
GG	158 (55.4)	160 (55.9)	1.00	100 (55.2)	88 (55.3)	1.00	58 (55.8)	72 (56.7)	1.00
GA	105 (36.8)	99 (34.6)	0.46 (0.17–1.26)	70 (38.7)	52 (32.7)	0.18 (0.05–0.64)	35 (33.7)	47 (37.0)	6.29E8 (0.00−)
AA	22 (7.7)	27 (9.4)	0.78 (0.38–1.60)	11 (6.1)	19 (11.9)	0.86 (0.33–2.24)	11 (10.6)	8 (6.3)	0.72 (0.23–2.34)
GA/AA	127 (44.5)	126 (44.0)	0.69 (0.36–1.33)	81 (44.8)	71 (44.6)	0.58 (0.24–1.37)	46 (44.23)	55 (43.3)	1.07 (0.34–3.33)
Allele			*P* a * = *0.816			*P* a * = *0.396			*P* a * = *0.526
G	421 (73.9)	419 (73.3)	1.00	270 (74.6)	228 (71.7)	1.00	151 (72.60)	191 (75.2)	1.00
A	149 (26.1)	153 (26.7)	1.03 (0.79–1.34)	92 (25.4)	90 (28.3)	1.16 (0.83–1.63)	57 (27.40)	63 (24.8)	0.87 (0.58–1.33)

a Two-sided test;

b ORs were adjusted for age, gender and lifestyle factors;

c ORs were adjusted for age and lifestyle factors.

To exclude environmental influence, we investigated the association between the potential three GC-related SNPs and the risk of GC in the age-matching population carrying health lifestyle (Table 7). One distinct difference was examined in terms of the genotype distribution of rs2072454 (*χ^2^* = 6.036, *P = *0.049). Specially, logistic regression analyses revealed that the TT genotype significantly increased the risk of GC in the population with daily eating breakfast (adjusted OR = 1.92, 95% CI = 1.07–3.44, *P*<0.05).

**Table 7:** Association between the three possible impact SNPs and the risk of GC in the age-matching population with health lifestyle factors.

SNPs	Eating on time		Average or not well salted food		Eating time >10 min		No smoking		No drinking		Daily eating breakfast	
	Cases	Controls	Cases	Controls	Cases	Controls	Cases	Controls	Cases	Controls	Cases	Controls
	N (%)	N (%)	N (%)	N (%)	N (%)	N (%)	N (%)	N (%)	N (%)	N (%)	N (%)	N (%)
**rs2072454**	N = 222	N = 257	N = 133	N = 221	N = 134	N = 159	N = 165	N = 203	N = 147	N = 178	N = 175	N = 259
CC	69 (31.1)	89 (34.6)	35 (26.3)	69 (31.2)	38 (28.4)	48 (30.2)	52 (31.5)	65 (32.0)	43 (29.3)	57 (32.0)	53 (30.3)	88 (34.0)
TC	110 (49.5)	135 (52.5)	74 (55.6)	123 (55.7)	72 (53.7)	91 (57.2)	83 (50.3)	111 (54.7)	77 (52.4)	97 (54.5)	85 (48.6)	139 (53.7)
TT	43 (19.4)	33 (12.8)	24 (18.0)	29 (13.1)	24 (17.9)	20 (12.6)	30 (18.2)	27 (13.3)	27 (18.4)	24 (13.5)	37 (21.1)	32 (12.4)
*P* a	0.145		0.363		0.445		0.419		0.474		0.049 b	
**rs1050171**	N = 211	N = 250	N = 125	N = 214	N = 127	N = 152	N = 159	N = 198	N = 141	N = 172	N = 163	N = 253
GG	157 (74.4)	171 (68.4)	93 (74.4)	147 (68.7)	93 (73.2)	103 (67.8)	120 (75.5)	131 (66.2)	114 (80.9)	119 (69.2)	119 (73.0)	174 (68.8)
GA	50 (23.7)	69 (27.6)	30 (24.0)	60 (28.0)	32 (25.2)	42 (27.6)	34 (21.4)	60 (30.3)	24 (17.0)	47 (27.3)	43 (26.4)	69 (27.3)
AA	4 (1.9)	10 (4.0)	2 (1.6)	7 (3.3)	2 (1.6)	7 (4.6)	5 (3.1)	7 (3.5)	3 (2.1)	6 (3.5)	1 (0.6)	10 (4.0)
*P* a	0.232		0.432		0.298		0.150		0.063		0.107	
**rs2293347**	N = 218	N = 252	N = 131	N = 217	N = 130	N = 156	N = 163	N = 199	N = 142	N = 174	N = 172	N = 254
GG	119 (54.6)	141 (56.0)	70 (53.4)	124 (57.1)	71 (54.6)	90 (57.7)	84 (51.5)	112 (56.3)	70 (49.3)	99 (56.9)	96 (55.8)	143 (56.3)
GA	81 (37.2)	87 (34.5)	55 (42.0)	76 (35.0)	48 (36.9)	52 (33.3)	64 (39.3)	73 (36.7)	58 (40.8)	62 (35.6)	61 (35.5)	87 (34.3)
AA	18 (8.3)	24 (9.5)	6 (4.6)	17 (7.8)	11 (8.5)	14 (9.0)	15 (9.2)	14 (7.0)	14 (9.9)	13 (7.5)	15 (8.7)	24 (9.4)
*P* a	0.788		0.278		0.818		0.590		0.382		0.948	

a two-sided test;

b OR = 1.92, 95% CI = 1.07–3.44, *P<*0.05 by logistic regression analyses adjusted for age, gender and lifestyle factors.

Exons not only encode the amino acid sequence of the protein, but also contain sequences that influence translation or mRNA degradation [19]. The loci were combined and subjected to haplotype inference analysis using the PHASE 2.0 program. There were four possible haplotypes in the total population and three possible haplotypes in the age-matching population, with a frequency of >4% (Table 8, Table 9). Compared to the GTTGCG haplotype, logistic regression analyses revealed that the ACAGCG haplotype was associated with a significantly decreased risk of GC (OR = 0.67, 95% CI = 0.49–0.92, *P*<0.05 adjusted for age, gender and lifestyle factors) in the total population (Table 8) rather than the age-matching population (OR = 0.83, 95% CI = 0.58–1.19, *P*>0.05 adjusted for age, gender and lifestyle factors) (Table 9). The other haplotypes were associated with a significantly decreased risk of GC (adjusted OR = 0.69, 95% CI = 0.52–0.90 for the total population; adjusted OR = 0.74, 95% CI = 0.56–0.99 for the age-matching population). Compared to the ACAGCG haplotype, the ACAACG could decrease the risk of GC (OR = 0.61, 95% CI = 0.40–0.94 adjusted for age, gender and lifestyle) in the total population, but the *P* value did not reach statistical significance by Bonferroni correction (Table 8).

**Table 8:** Association study with haplotypes consisting of pairwise combination of six SNPs between cases and controls in the total population.

Haplotypes^a^	Allele frequencies				OR (95%CI)^b^	OR (95%CI)^b^	OR (95%CI)^b^	OR (95%CI)^b^
	Cases		Controls					
	N	%	N	%				
G T T G C G	236	30.5	188	24.0	1.00			
A C A G C G	110	14.2	131	16.7	0.67 (0.49–0.92)^c^	1.00		
A C A G C A	169	21.8	179	22.8	0.75 (0.57–1.00)^c^	0.89 (0.64–1.24)	1.00	
A C A A C G	63	8.1	59	7.5	0.85 (0.57–1.27)	0.61 (0.40–0.94)^d^	0.88 (0.59–1.34)	1.00
Others	196	25.3	227	29.0	0.69 (0.52–0.90)^b^	0.75 (0.55–1.03)	1.09 (0.82–1.45)	1.24 (0.83–1.85)

a The sequence of the SNPs in the Haplotypes was rs2227983, rs2072454, rs17337023, rs1050171, rs1140475 and rs2293347;

b ORs were adjusted for age, gender and lifestyle factors;

c *P<*0.05;

d *P*>0.05 by Bonferroni correction.

**Table 9:** Association study with haplotypes consisting of pairwise combination of six SNPs between cases and controls in the age-matching population.

Haplotypes^a^	Allele frequencies				OR (95%CI)^b^	OR (95%CI)^b^	OR (95%CI)^b^
	Cases		Controls				
	N	%	N	%			
G T T G C G	189	32.1	156	26.5	1.00		
A C A G C A	117	19.9	129	21.9	0.75 (0.54–1.04)	1.00	
A C A G C G	89	15.1	89	15.1	0.83 (0.58–1.19)	0.91 (0.62–1.33)	1.00
Others	193	32.8	214	36.4	0.74 (0.56–0.99)^c^	1.01 (0.73–1.38)	1.11 (0.78–1.58)

a The sequence of the SNPs in the Haplotypes was rs2227983, rs2072454, rs17337023, rs1050171, rs1140475 and rs2293347;

b ORs were adjusted for age, gender and lifestyle factors;

c *P<*0.05.

## Discussion

Although a great number of new GC cases were seen throughout the world in the latest decades, the exact mechanisms underlying gastric carcinogenesis are not yet fully understood. Similar to previous research [20], [21], our result showed that the men generally have been developing GC twice as frequently as women in China. It was suggested by Michael *et al.* that much of the global variation in cancer incidence has been attributed to environmental influences, including dietary preferences and unhealthy lifestyle factors [22]. In the present study, therefore, we were interested to test whether some unhealthy lifestyle factors could increase the risk of GC with gender differences in China. Six lifestyle factors, including regularly taking meals, preference for salty food, eating time, smoking status, drinking status, and eating breakfast, were identified to be influenced the risk of GC with gender differences, which was consistent with the previous studies in east China [20], [23], [24]. Especially, preference for salty food, drinking and smoking were the strongest and most consistent risk factors for GC.

Resent researches suggested that a high intake of salt (sodium) could increase the risk of GC [21], [24]–[26]. It was also evidenced by our observation that the GC patients in Jiangsu province were preference for salty foods, such as salted meat, pickled vegetables, and pickled vegetable juice, which might be contaminated by N-nitroso compounds. However, the N-nitroso compounds were the most frequently proposed related to the increased risk of upper-gastrointestinal cancers [27]. Drinking and smoking, another two dominant risk factors for GC in the world [28], were also examined in this study. We found a significant association between drinking and GC risk in Jiangsu province. It was evidence by a laboratory study that smoking could increase the apoptosis in the rat gastric mucosa by an increase in XO activity [29], and alcohol could also exert influence on acid secretion, gastric emptying, and certain acid-related diseases, such as gastritis accompanied with damage of the gastric mucosa, and the following inflammatory reaction will in turn promote gastric cell proliferation and differentiation [30]. In the process, the N-nitroso compounds and mycotoxins from some salty food may induce gene mutations [31], [32], thus preference for salty food may be the original risk of GC. And evidence pointed to an association with pathways involved in developmental processes [33]. Key molecules of these pathways were the receptor tyrosine kinases, which were found to be aberrantly activated or overexpressed in a variety of tumors and therefore represent promising targets for therapeutical intervention [13].

EGFR was one of the key molecules, and many lines of evidence suggest that highly invasive GC is associated with the aberrant activation or overactivation of EGFR due to gene amplification or structural alterations [13]. Very recently, a case-control study of 61 cases and 20 controls in Henan province, located in middle China, showed that the EGFR rs28384375 C/T polymorphism may promote the occurrence and development of GC [34]. Moreover, another case-control study of 138 cases and 170 controls in Jiangxi province, located in south-east China, revealed that the EGFR rs763317 G/A polymorphism may associate with an increased risk of GC [35].

EGFR is a growth factor receptor tyrosine kinase and belongs to the receptor tyrosine kinase superfamily, whose members are characterized by an extracellular domain (where ligand binding ligands takes place), a short lipophilic transmembrane domain, and an intracellular domain that harbors the tyrosine kinase activity [36]. EGFR can be activated by binding ligands, such as EGF and TGF-α, and it plays pivotal roles in development, proliferation and differentiation. The activation of EGFR may contribute to the transformation of cellular phenotypes and provide tumor cells with substantial growth and survival advantages [37]. Many human tumors exhibit EGFR overexpression, which is correlated with an advanced tumor stage or a poor clinical outcome, such as non-small cell lung cancer [38], colorectal cancer [39], breast cancer [40], head and neck cancer [41], bladder cancer [42], and GC [43].

There is currently increasing interest in SNP mutations in EGFR, given that they could affect the efficacy of EGFR tyrosine kinase inhibitor (TKI) treatment in various cancers [44], colorectal cancer [39], non-small cell lung cancer [45], GC [46]–[49]. It is well known that exons not only encode the amino acid sequence of the protein, but also contain sequences that influence translation or mRNA degradation [19]. Thus, EGFR exon SNPs could influence EGFR gene expression and/or protein activity and thereby alter the affinity of EGFR for not just its ligands, but also for anticancer agents that target this protein. Indeed, Puyo *et al.* found that mutations in exons 18–21 of EGFR enhance the activity of TKIs, while the deletion of exons 2–7 is associated with glioblastoma oncogenesis [49]. Moreover, two clinical studies showed that SNPs in EGFR exons 18, 19, 21, and 25 affect the clinical efficacy of gefitinib and may be potential biomarkers for the prediction of the clinical outcome of gefitinib-treated patients with advanced non-small cell lung cancer (NSCLC ) [50], [51].

In the present study, the six exons SNPs in EGFR were slightly associated with the risk of GC both in the total population and the age-matching population even with gender differences. It was noted that the SNP rs2072454 was significantly associated with the risk of GC. Especially, the variant rs2072454 T allele and TT genotype were associated with an increased risk of GC. However, the rs2227983, rs17337023, rs1050171 and rs1140475 SNPs did not relate with GC risk. With regard to the missense locus rs28384375, only the T allele was detected in cases and controls. Many reports suggested that several EGFR SNPs, including rs2227983 (also designated as Arg521Lys or R497K) [39], [52], [53], rs1050171 (also designated as Q787Q) [54], [55] and rs2293347 (C2982T) [56], were more likely to affect the biological behavior of tumors (such as tumor growth, invasion, metastasis, and progression) than to define susceptibility to cancer development. Since EGFR, a key mediator of angiogenesis, can regulate its target genes such as VEGF, which can directly affect tumor biological behavior. These results would also explain why EGFR SNPs can serve as key determinants of a response to EGFR TKI-based chemotherapy [11]. Therefore, these gene mutations may confer the complexity and embarrassment for GC treatment and survival [57].

When Puyo *et al*. [49] analyzed the association between specific EGFR functional polymorphisms and anticancer drug activity in 60 human tumor cell lines established by the National Cancer Institute, the frequency of the nonsynonymous SNP rs28384375 (also designated as Val592Ala) was 0.5, while the heterozygous frequency of the −216G>T SNP (also designated as rs287129) was 0.346. The cell lines that were heterozygous and variant homozygous for the −216 G>T SNP showed significantly higher expression of the EGFR gene than the homozygous wild-type lines. Moreover, compared with cell lines without a variant allele, the cell lines with at least one variant T allele at the −216 G>T SNP were more sensitive to erlotinib and less sensitive to geldanamycin, topoisomerase I and II inhibitors, and alkylating agents. Interestingly, our results showed that the GTTGCG haplotype was more prevalent in GC cases than in cancer-free controls, and that the ACAGCG haplotypes were associated with a significantly decreased risk of GC (adjusted OR = 0.67, 95% CI = 0.49–0.92 for ACAGCG, adjusted OR = 0.69, 95% CI = 0.52–0.90 for others) in the total population (Table 8). However, in the age-matching population, no significant association was examined between ACAGCA haplotype and risk of GC (adjusted OR = 0.83, 95% CI = 0.58–1.19) (Table 9). Thus, the T alleles of rs2072454 and rs17337023, especially the former, might be associated with the risk of GC. Therefore, combined analysis of the six SNPs, especially the T alleles of rs2072454 and rs17337023, may be useful for predicting the risk of GC.

Several limitations in the present study need to be addressed: 1) its sample size may not have been large enough to detect SNPs with low variant frequency, such as rs28384375; 2) the polymorphisms that were investigated in this study were selected on the basis of their effects on EGFR function and may not give a comprehensive view of the genetic variability in EGFR exons; 3) detailed information about the GC cases was not collected, including patient survival, whether the tumors were the intestinal or diffuse type, whether there was metastasis, and what the effect of drug therapy was.

In conclusion, the present study suggested that the differences of lifestyle between males and females might be as the reason of higher incidence rates in males than those in females. Although only one SNP (rs2072454) was significantly associated with an increased risk of GC, combined analyzing the other six EGFR exon SNPs together may be useful for predicting the risk of GC. Further studies are warranted to establish these findings and to address the underlying mechanisms.

## Supporting Information

Figure S1 **The fluorescent products of LDR were differentiated by ABI sequencer 377 for the seven SNPs in EGFR exons.** In total, more than 90% of the products were successfully differentiated by ABI sequencer 377.
(TIF)

Table S1 **The PCR primer sequences for the seven loci in the EGFR gene.** The primers were designed by Primer Premier 6 software and synthesized by Shanghai Sangon Biological Engineering Technology and Services.
(DOC)

Table S2 **Probe sequences of the seven SNPs in EGFR gene.** The primers were synthesized by Shanghai Sangon Biological Engineering Technology and Services.
(DOC)
