# Supplementary figures and images for: Association among Polymorphisms in EGFR Gene Exons, Lifestyle and Risk of Gastric Cancer with Gender Differences in Chinese Han Subjects
Source: PLoS One. 2013 Mar 29;8(3):e59254. doi: 10.1371/journal.pone.0059254 (PMC3612075; doi:10.1371/journal.pone.0059254)

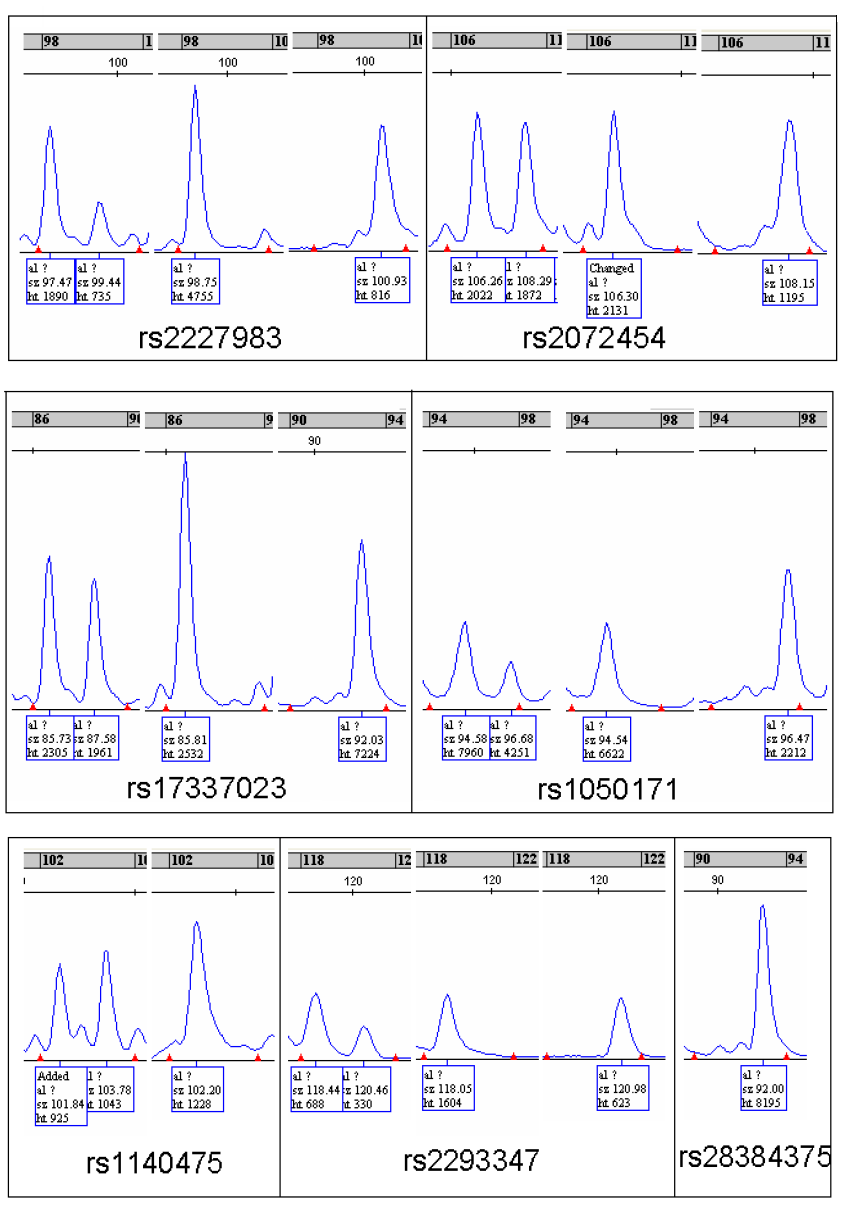

Supplement: Figure S1 — The fluorescent products of LDR were differentiated by ABI sequencer 377 for the seven SNPs in EGFR exons. In total, more than 90% of the products were successfully differentiated by ABI sequencer 377. (TIF) [file pone.0059254.s001.tif]
