# Supplementary material for: Association among Polymorphisms in EGFR Gene Exons, Lifestyle and Risk of Gastric Cancer with Gender Differences in Chinese Han Subjects
Source: PLoS One. 2013 Mar 29;8(3):e59254. doi: 10.1371/journal.pone.0059254 (PMC3612075; doi:10.1371/journal.pone.0059254)
Supplement: Table S1 — The PCR primer sequences for the seven loci in the EGFR gene. The primers were designed by Primer Premier 6 software and synthesized by Shanghai Sangon Biological Engineering Technology and Services. (DOC) [file pone.0059254.s002.doc]

**Table S**1 The PCR primer sequences for the seven loci in the EGFR gene

| **SNPs** | **Forward** | **Reverse** | **Length** |
| --- | --- | --- | --- |
| rs2227983 | TGCTGTGACCCACTCTGTCT | CAACGCAAGGGGATTAAAG | 194 |
| rs17337023 | ACCACCAATCCAACATCCAG | CCACAGCAGTGTGGTCATTC | 250 |
| rs1140475 | GAAGCAAATTGCCCAAGACT | AGGCGTTCTCCTTTCTCCAG | 237 |
| rs2293347 | AGAGAGCTCAGGAGGGGAGT | TAGCATCTCTACGGGCCATT | 216 |
| rs2072454 | AAAGAGTGCTCACCGCAGTT | CATAGGAGCTGGAGGCAGAG | 213 |
| rs28384375 | GCATGAACATTTTTCTCCACCT | CTCACCCGTAGGTGCAGTTT | 195 |
| rs1050171 | CTCCAGGAAGCCTACGTGAT | TTATCTCCCCTCCCCGTATC | 230 |
