# Supplementary material for: Association among Polymorphisms in EGFR Gene Exons, Lifestyle and Risk of Gastric Cancer with Gender Differences in Chinese Han Subjects
Source: PLoS One. 2013 Mar 29;8(3):e59254. doi: 10.1371/journal.pone.0059254 (PMC3612075; doi:10.1371/journal.pone.0059254)
Supplement: Table S2 — Probe sequences of the seven SNPs in EGFR gene. The primers were synthesized by Shanghai Sangon Biological Engineering Technology and Services. (DOC) [file pone.0059254.s003.doc]

**Table S2 Probe sequences of the seven SNPs in EGFR gene**

| **SNPs** | **Probe** | **Probe sequence (5’ - 3’)** | **LDR length** |
| --- | --- | --- | --- |
| rs2227983 | modify | P-TGGGCTCCGGGCCCCAGCAGCCCTCTTTTTTTTTTTTTTTTTTTTTTTT-FAM |  |
|  | A | TTTTTTTTTTTTTTTTTTTTTTTTACATTCCGGCAAGAGACGCAGTCCT | 98 |
|  | G | TTTTTTTTTTTTTTTTTTTTTTTTTTACATTCCGGCAAGAGACGCAGTCCC | 100 |
| rs17337023 | modify | P-GTGCATCTGTAGGAAGTGAAAGAGATTTTTTTTTTTTTTTTTT-FAM |  |
|  | A | TTTTTTTTTTTTTTTTTTTGGACAGCCTTCAAGACCTGGCCCT | 86 |
|  | T | TTTTTTTTTTTTTTTTTTTTTGGACAGCCTTCAAGACCTGGCCCA | 88 |
| rs1140475 | modify | P-GTCACCCCTGAGAGGATGAAGCAAGTTTTTTTTTTTTTTTTTTTTTTTTTT-FAM |  |
|  | C | TTTTTTTTTTTTTTTTTTTTTTTTTTTCCAAAGGTCATCAACTCCCAAACG | 102 |
|  | T | TTTTTTTTTTTTTTTTTTTTTTTTTTTTTCCAAAGGTCATCAACTCCCAAACA | 104 |
| rs2293347 | modify | P-TCCAACTTCTACCGTGCCCTGATGGTTTTTTTTTTTTTTTTTTTTTTTTTTTTTTTTTT-FAM |  |
|  | A | TTTTTTTTTTTTTTTTTTTTTTTTTTTTTTTTTTAATGCATTTGCCAAGTCCTACAGAT | 118 |
|  | G | TTTTTTTTTTTTTTTTTTTTTTTTTTTTTTTTTTTTAATGCATTTGCCAAGTCCTACAGAC | 120 |
| rs2072454 | modify | P-TTGCACAGGGCAGGGTTGTTGCTGATTTTTTTTTTTTTTTTTTTTTTTTTTTT-FAM |  |
|  | C | TTTTTTTTTTTTTTTTTTTTTTTTTTTTGTCCCGCCACTGGATGCTCTCCACG | 106 |
|  | T | TTTTTTTTTTTTTTTTTTTTTTTTTTTTTTGTCCCGCCACTGGATGCTCTCCACA | 108 |
| rs28384375 | modify | P-CGCAGTGGGGGCCGTCAATGTAGTGTTTTTTTTTTTTTTTTTTTT-FAM |  |
|  | C | TTTTTTTTTTTTTTTTTTTTATGACTCCTGCCGGGCAGGTCTTGG | 90 |
|  | T | TTTTTTTTTTTTTTTTTTTTTTATGACTCCTGCCGGGCAGGTCTTGA | 92 |
| rs1050171 | modify | P-TGCACGGTGGAGGTGAGGCAGATGCTTTTTTTTTTTTTTTTTTTTTT-FAM |  |
|  | A | TTTTTTTTTTTTTTTTTTTTTTGAAGGGCATGAGCTGCGTGATGAGT | 94 |
|  | G | TTTTTTTTTTTTTTTTTTTTTTTTGAAGGGCATGAGCTGCGTGATGAGC | 96 |
